# Interpretation of a Quantitative Diagnosis Model of Traditional Chinese Medicine Syndromes Based on Computer Adaptive Testing

**DOI:** 10.1155/2022/3203158

**Published:** 2022-06-30

**Authors:** Simeng Yao, Zhongyu Huang, Xianhua Liu, Qiaofeng Yan, Jing Tang, Fengbin Liu, Zhengkun Hou

**Affiliations:** ^1^Guangzhou University of Chinese Medicine, Guangzhou, Guangdong, China; ^2^Integrated Chinese and Western Medicine Postdoctoral Research Station, Jinan University, Guangzhou, Guangdong, China; ^3^The First Affiliated Hospital of Guangzhou University of Chinese Medicine, Guangzhou, Guangdong, China; ^4^Laboratory for Neuroscience in Health and Disease, Guangzhou First People's Hospital, School of Medicine, South China University of Technology, Guangzhou, Guangdong, China; ^5^Shenzhen Baoan Traditional Chinese Medicine Hospital Group, Shenzhen, Guangdong, China; ^6^No. 1 Traditional Chinese Medicine Hospital Changde, Changde, Hunan, China; ^7^Department of Traditional Chinese Medicine, General Hospital of the Western Theater Command, Chengdu, Sichuan, China

## Abstract

**Objectives:**

The aim of this study is to interpret a quantitative diagnosis model of traditional Chinese medicine (TCM) syndromes based on computer adaptive testing (CAT), from the perspective of both patients and clinicians.

**Methods:**

In this cross-sectional study, patients with postprandial distress syndrome completed the CAT model of TCM syndromes and the Chinese version of the Quality of Life Questionnaire for Functional Digestive Disorders (Chin-FDDQL); the clinicians' diagnosis was concurrently recorded. The patients completed this questionnaire again after 14 ± 2 days. The kappa test and paired chi-square test were used to evaluate the consistency between the CAT model and clinical diagnosis. Minimal clinically important differences (MCID) of the Chin-FDDQL scores were used to assess clinical efficacy from the patients' perspective. Logistic regression was used to examine the association between changes in the CAT model syndrome domain scores and changes in clinical outcomes.

**Results:**

Changes in the CAT model syndrome domain scores may affect the clinical outcomes of patients with the total scores of Chin-FDDQL (all *P* < 0.05). There was a correlation between changes in the CAT model syndrome domain scores and the patients' clinical outcomes. Different syndrome elements had different effects on various Chin-FDDQL domains, which was consistent with the theory of TCM.

**Conclusions:**

This study proposes a method for the clinical interpretation of the CAT model of TCM syndromes, including evidence derived from the application. It may provide a reference for future interpretation of other CAT models.

## 1. Introduction

Accurate syndrome diagnosis is the foundation of effective management and treatment [1]. However, traditional approaches to syndrome diagnosis rely on clinicians' experience; at present, there is a lack of objective traditional Chinese medicine (TCM) syndrome detection protocols [2]. Therefore, the use of statistical models and artificial intelligence, among other methods, has increased in recent years, aiming to make TCM syndrome differentiation objective [3, 4].

We have previously introduced syndrome elements based on the theory of TCM and established a quantitative diagnosis model of TCM syndromes in functional gastrointestinal diseases, specifically, functional dyspepsia and irritable bowel syndrome, using the traditional statistical theory and modern advanced measurement theory [2, 5]. The model allows patients to input their symptoms and obtain scores per syndrome domain, helping to quantify the syndrome; subsequently, the model has been combined with computer technology. The computer adaptive test (CAT) model [6] streamlines the process of patients inputting their symptoms and maintains accuracy in syndrome differentiation. It is a novel and feasible tool for the quantification of TCM syndromes.

However, the clinical interpretation of the model has not been examined to-date. To our knowledge, a clinical interpretation of the quantitative CAT model of TCM syndromes has not been established for any specific disease. In fact, the accuracy of syndrome differentiation is often based on a clinician's judgment, which is unsatisfying. Patients are the main recipient of syndrome differentiation. The TCM theory stipulates that changes to any of the syndrome domains may change patients' symptoms and outcomes. Therefore, we examined the changes in patients' symptoms and clinical outcomes to assess whether the CAT model-based syndrome differentiation is accurate, helping in the quantification and objective assessment of TCM syndromes.

In this study, we used a postprandial distress syndrome (PDS) model. PDS is among the most common functional gastrointestinal diseases observed in clinical practice, and its incidence is increasing. PDS is not life-threatening; however, it is associated with a long disease course and recurring symptoms, which may affect the patients' quality of life. PDS is also associated with a high economic burden to patients and healthcare systems [7]. It can be divided into two subtypes: PDS and epigastric pain syndrome (EPS). Impaired gastric accommodation is more prevalent in PDS than in EPS [8]. PDS belongs to the TCM category of gastric stuffiness and is among the most common diseases in the clinic. TCM has been reported as an effective complementary and alternative approach in the treatment of PDS [9, 10].

There are no laboratory indicators that evaluate clinical outcomes of PDS. TCM tends to account for patients' subjective symptoms; therefore, we converted patients' symptoms into numeric values, using the Chinese version of the Quality of Life Questionnaire for Functional Digestive Disorders (Chin-FDDQL) [11], which is a commonly used patient outcome reporting scale; minimal clinically important differences (MCID) were calculated to estimate any relationship between the changes in the Chin-FDDQL scores and clinically meaningful outcomes for patients. The MCID may help make symptom and outcome reporting more objective [12]. It is commonly used in the clinical interpretation of patient-reported outcomes and can be calculated using anchor- and distribution-based methods [13–16].

## 2. Methods

This cross-sectional study included patients who attended the outpatient clinic at the study site. This work was approved by the Clinical Research and Ethics Committee at the First Affiliated Hospital of the Guangzhou University of Chinese Medicine (NO. K (2019) 074), and all patients were diagnosed by senior clinicians referring to the Roman IV classification criteria for PDS.

Patients were eligible for the present study if they met the following criteria: aged ≥16 years, met the Rome IV PDS criteria, and agreed to study participation. Patients were excluded from the present study if they had other digestive system diseases, cognitive or other impairments (including mental illness and visual impairment, among others) that affected their ability to complete self-reports, or diagnoses of cardiovascular or cerebrovascular diseases, renal insufficiency, hematopoietic system, or another serious primary disease; pregnant women were also excluded from the present study. Further, data from patients that met the following criteria were considered “invalid” and were excluded from analysis: misdiagnosis, another diagnosis, or a major accident experienced during the study period, loss to follow-up, or missing ≥20% of data.

### 2.1. Data Collection

The CAT model is an adaptive quantitative evaluation system (patent no.: 2017 sr559575) for TCM syndromes of FGIDs, covering three TCM diseases: stomachache, gastric stuffiness, and diarrhea. It integrates the TCM syndrome differentiation diagnosis tree, artificial intelligence, computer engineering, and multivariate statistical models that account for syndrome domains and other aspects of the TCM theory. Development, simulation, and verification of the CAT model have been previously described [6, 17–19]. In this study, we selected a common PDS disease, which belongs to the gastric stuffiness category of TCM, to explore the CAT model clinical interpretation methods.

The gastric stuffiness CAT model had 39 items extracted from a bank of 215 items. It used the maximum determinant value of the information matrix to select the next test item; in addition, the maximum a posteriori capability level assessment estimates were used. There were 20 answers available as the test termination condition. We asked patients to input data on their symptoms and experiences into the CAT evaluation system. Finally, the patients' scores per syndrome domain were displayed in the form of a radar chart.

The Chin-FDDQL [11] was translated by our team from the original version, designed to measure the pathology and symptom scores of FD and irritable bowel syndrome across eight domains (daily activity, anxiety, diet, sleep, discomfort, health perceptions, stress levels, and total scores) and 43 items [20]. It is a useful health assessment instrument for Chinese patients with FD; it is associated with good reliability, validity, responsibility, item test function, differential item functioning characteristics, and interpretation systems [19, 20].

Outcome assessment and follow-up protocols were as follows. First, the investigators presented the study aims to eligible patients; subsequently, the patients completed the Chin-FDDQL, using the Wen Juan Xing application, and the CAT model system; the questionnaires were completed again after 14 ± 2 days. The clinicians' diagnoses were recorded at the same time; for patients unable to attend follow-up assessments on schedule, we provided a link to the electronic version of the scale via WeChat or we collected their answers via phone interviews, subsequently requesting that the participating clinicians make a diagnosis based on the patient's statement.

### 2.2. Statistical Methods

The CAT model and Chin-FDDQL data were exported to and sorted in Excel. To standardize the evaluation of syndrome domain, the CAT model scores were transformed, according to the distribution characteristics of the full-sample computer adaptive test scores. The conversion formula was as follows:

(1) $$\begin{array}{c} {\text{Score}}_{\text{standard}}=\frac{{\text{Score}}_{\text{CAT}}-{\text{Score}}_{\text{min}}}{{\text{Score}}_{\text{max}}-{\text{Score}}_{\text{min}}}\times 100. \end{array}$$

The clinicians' syndrome differentiation results were divided into syndrome element forms, according to the theory of syndromes, and used as state variables. The CAT model diagnosis results were used as test variables to draw the receiver operating characteristic (ROC) curve for every syndrome element. The area under the curve (AUC) was used to verify the accuracy of model diagnosis; AUC values of >0.8 were considered indicative of high model accuracy. The Youden Index was used as a reference parameter; when the Youden Index reached its maximum value, the score corresponding to the cut-off point was regarded as the diagnostic threshold of an element.

We examined the CAT model diagnosis from the physician's perspective, according to the diagnostic threshold of every syndrome domain. We then used the kappa test and paired chi-square test to analyze the consistency between the CAT model and expert diagnoses. Kappa values of ≥0.75 indicated excellent consistency; those 0.40–0.75 and <0.40 represented fair to good and poor consistency, respectively.

To account for the patients' perspective, we used the paired sample *t*-test or Wilcoxon signed-rank test to measure the responsiveness of the Chin-FDDQL scores to time-dependent changes. We then calculated the associated MCID. To reduce bias associated with using a single method, we obtained averages of the MCID values by anchor-based and distribution-based methods; these values were used as final estimates. Anchor-based methods rely on an external measure of change as the standard, and distribution-based methods are based on a statistical measure of variability.

Because PDS has no objective index for clinical efficacy evaluation, we chose the most applied patient self-assessment method, adding an item as an anchor at the end of the Chin-FDDQL. This item was captured during the follow-up period to determine the MCID [21]. This item was “how do you feel now compared with last time?,” with the following response options: obviously worse, somewhat worse, no change, somewhat better, and obviously better; the corresponding scores were set to −2, −1, 0, 1, and 2 points, respectively. We identified patients who reported having experienced a change and then calculated the difference between their baseline and follow-up Chin-FDDQL scores (total and domain-specific). If the score difference values obeyed the normal or skewed distribution, the mean or median of the difference was used as the MCID value, respectively.

This study used the common effect size (ES) estimating methods [21]; MCID was estimated by multiplying the baseline standard deviation value of the Chin-FDDQL scores by the ES. Some studies in China have proposed an ES value of 0.5 [22], while recommending ES values of 0.2 for the evaluation of the MCID in the Western context [23]. Therefore, we used both methods to estimate the MCID; we combined these estimates with the expert opinion to obtain the MCID that reflected clinical practice.

**Figure 1:**
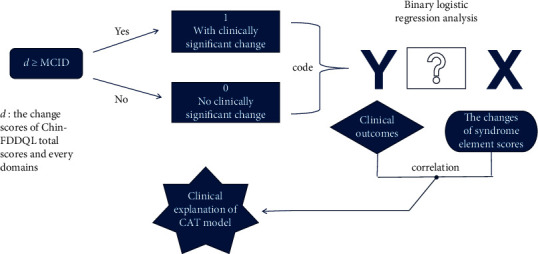
Schematic diagram of exploring the clinical explanation of the CAT model.

To explore the clinical value of the CAT model, we compared changes (*d*) to the Chin-FDDQL total and domain scores with the corresponding MCID; *d* ≥ MCID represented clinical benefits from the patients' perspective. We then classified patient outcomes into “change” and “no change” groups. Finally, we performed logistic regression analysis to explore the association between syndrome element score changes in the CAT model (independent variable) and clinical outcomes (dependent variable) (1 = clinically significant change, 0 = no clinically significant change).  Figure 1 presents a schematic of the approach to the CAT model exploration.

## 3. Results

A total of 300 patients with PDS were included in the present study at baseline, and a total of 291 patients were included at follow-up, with a total of nine study dropouts. The patients' demographic characteristics are presented in Table 1. There were slightly more females than males; most patients were young and middle-aged, and the proportion of those with a bachelor's or higher degree was relatively high.

Syndrome element scores included in the CAT model are presented in Table 2. Although the average syndrome element scores decreased over time, this change was not uniform; specifically, liver and qi stagnation syndrome element scores changed markedly, while spleen-dampness and stomach syndrome element scores changed the least. The AUC for the CAT model was >0.8 (Appendix I). The Youden Index values for liver, stomach, spleen-dampness, qi deficiency, heat, and qi stagnation diagnostic thresholds were 39, 44, 52, 41, 47, and 43 points, respectively.

The McNemar test findings of the qi deficiency syndrome element (*P* < 0.05) differed between the CAT and clinician diagnoses. The kappa coefficient was 0.628, indicating diagnostic consistency; however, the kappa coefficient was <0.75 for the general diagnostic consistency. For the other five syndromes, the McNemar test result was nonsignificant, indicating consistency between the diagnoses obtained by the CAT model and those obtained by clinicians. The kappa test revealed moderate consistency between the two diagnoses; the heat syndrome was associated with the highest kappa coefficient. The total scores of the Chin-FDDQL did not obey the normal distribution; the Wilcoxon test finding revealed *P*values of <0.05, indicating sensitivity of the Chin-FDDQL score to any changes in patients' conditions.

Using the patients' experience as anchors, we identified 198 patients reporting changes in their condition, including 39, 156, and 3 patients that experienced obvious and some improvement, and a worsening of their condition, respectively. The differences in scores were normally distributed, and the average value was used as the MCID. However, PDS is a recurrent chronic disease, with long courses of treatment; given the study period of 14 ± 2 days, no obvious changes from baseline were observed. In our previous study, the total Chin-FDDQL score change was approximately 4 points (minimum clinically significant change) [24]. Therefore, in the present study, we used the median MCID as the total score. The differences in domain scores followed a skewed distribution; thus, the median value was used; however, the median scores in the diet and stress domains were 0 points, which were inconsistent with actual clinical. Considering the possible bias of sample, we combined expert opinion method and took the average as MCID in diet and stress domains.

Given that the study period was short, the changes between baseline and follow-up scores were small and combined with expert clinical experience. We used an ES2 of 0.2 as the end result of distribution-based methods. The MCID results obtained by distribution-based, anchor-based, and weighting methods are shown in Table 3. The final MCID results are determined by the weighting methods.

According to the results, the total scores of the Chin-FDDQL must be changed at least 4.5485 to represent clinically meaningful improvements. Among various domains, the MCID values of worry (6.9477) and disease control (6.2919) were high, and those of daily activities (3.2043) and stress (3.5209) were low, suggesting that anxiety and disease control scores require greater changes than do daily activity and stress scores for patients to experience clinical benefits.

Tables 4–12 present findings on the association between changes in syndrome element scores of the CAT model and the patients' clinical outcomes, suggesting that score change in any syndrome element may affect patient outcomes; changes to the spleen-dampness scores had the greatest impact on patient outcomes.

**Figure 2:**
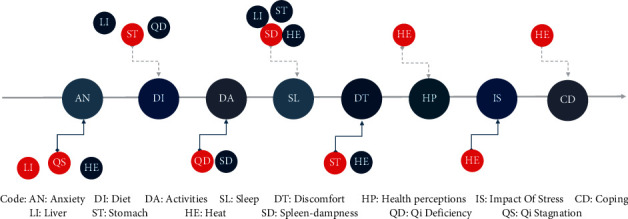
The influence of different syndrome element on clinical outcomes in various domains are different.

Meanwhile, different syndrome elements of the CAT model had a differential impact on various domains of the Chin-FDDQL score. For example, changes to qi deficiency scores had a great influence on the daily activity scores (*P* < 0.001). Changes to stomach scores significantly affected discomfort (<0.001) and diet (*P*=0.02) outcomes. Health perceptions (*P*=0.002), coping abilities (*P* < 0.001), discomfort (*P*=0.001), stress (*P*=0.023), and anxiety (*P*=0.024) scores were correlated with the changes to heat scores. Changes to spleen-dampness (*P* < 0.001), liver (*P*=0.025), stomach (*P*=0.023), and heat (*P*=0.012) scores affected sleep (Figure 2).

## 4. Discussion

Syndrome diagnosis is at the core of TCM prescriptions. However, there is no standardized approach to syndrome diagnosis, which may restrict TCM modernization. Quantitative models integrated with modern technologies such as artificial intelligence may provide novel instruments for the objectification of TCM syndrome assessment. Despite a growing number of available models, the clinical interpretation of the model has not been established to date, resulting in the model not being used in clinical practice.

In this study, we used the conventional method to evaluate the reliability of the model diagnosis by considering the clinician syndrome differentiation results as the gold standard; AUC values were >0.8, indicating good diagnostic accuracy of the model (*P* < 0.05). Nevertheless, this finding suggests that the model may be further optimized, likely by adding factors such as tongue and pulse diagnosis. This study focused on a new approach from the perspective of patients to interpret the model. The findings obtained by conventional methods are presented in the appendix.

The aim of this study was to establish a relationship between the CAT model findings and clinical practice. The diagnosis of a syndrome is made by clinicians, based on a set of patients' symptoms and signs; the TCM theory postulates that symptom changes may affect syndrome elements, suggesting a correlation between changes to the model syndrome element scores and clinically meaningful outcomes. Based on model score changes, clinicians may track patient symptom changes, providing evidence for the efficacy of TCM. However, to our knowledge, there is currently no objective method to assess the relationship between subjective symptoms and syndrome characteristics.

A scale is a commonly used clinical instrument to measure the disease status that cannot be accurately quantified. This study used the Chin-FDDQL to measure patients' subjective symptoms, and the MCID was used to correlate the Chin-FDDQL scores with clinical outcomes. The relationships between the model score changes and patient outcomes were quantified, aiding in an objective interpretation of the model. The MCID refers to the minimum change in scores that a patient considers beneficial, regardless of the associated side effects or costs [12]. It has been used in the clinical interpretation of scales related to computer adaptive tests [25–27]; however, a TCM syndrome quantification model remains to be established.

In this study, we applied the MCID to the clinical interpretation of the TCM syndrome quantification model, showing that any syndrome element score change may affect clinical outcomes. In addition, we found that different syndrome elements of the CAT model differentially affected the Chin-FDDQL domains, in a manner consistent with that proposed by the TCM theory; for example, stomach scores may significantly affect discomfort and diet outcomes, both of which are associated with gastrointestinal complaints.

This study has two main limitations. First, the study period was relatively short, and the captured score changes were small; consequently, this study showed a relationship between score changes and patient outcomes but did not establish a specific regression equation. Future studies should involve extended follow-up to explore the contribution of every syndrome element to the changes in clinical outcomes and to establish the corresponding regression equation to improve syndrome quantification. Second, despite the use of multiple methods to estimate the MCIDs, the presented values may be subject to bias. The strengths of this study include objective evaluation of the accuracy of syndrome differentiation by the CAT model, and a description of a novel method for the interpretation of a quantitative diagnosis of a TCM syndrome.

## 5. Conclusion

This study showed an association between changes to the syndrome element scores of the CAT model and patient outcomes. In addition, this study showed that changes to different syndrome elements had a differential impact on the Chin-FDDQL sub-scores; this finding is consistent with the theory of TCM, which indicates that the MCID values of the relevant quality of life scales may aid the clinical interpretation of the CAT model of a TCM syndrome. These findings may provide a reference for interpretation of other CAT models.

## 6. Disclosure

Simeng Yao and Zhongyu Huang contributed equally to this article as co-first authors.

## Figures and Tables

**Table 1:** Demographic characteristics of 291 patients with postprandial distress syndrome.

	Respondents	Total (*n* = 291)	Percentage (%)
Gender	Male	139	47.8
	Female	152	52.2

Level of education	Primary	9	3.1
	Junior high	62	21.3
	Senior high	72	24.7
	Junior college	33	11.3
	Bachelor or above	115	39.5

Age	16–30	59	20.3
	31–45	126	43.4
	≥45	106	36.4

Occupation	Trading	92	31.6
	Civil	58	19.9
	Servant	71	24.4
	Industry	31	10.7
	Student	33	11.3
	No data	6	2.1

**Table 2:** Diagnostic scores of the CAT model.

Syndrome element	Baseline		Follow-up	
	Average	Standard deviation	Average	Standard deviation
Liver	40.7468	8.5975	36.5049	8.3375
Stomach	47.5526	9.0654	47.1390	8.8166
Heat	45.2516	12.0908	44.2993	11.4685
Spleen-dampness	54.5902	8.0923	54.1714	7.7693
Qi deficiency	38.6171	10.6268	38.0749	10.8143
Qi dtagnation	46.3420	10.7829	44.4857	10.4074

**Table 3:** MCID results obtained by three methods.

Domain	Distribution-based methods (50%)	Anchor-based methods (50%)	Weighting methods
Activities	3.2835	3.1250	3.2043
Anxiety	3.8954	10.0000	6.9477
Diet	3.8171	3.4301	3.6236
Sleep	3.9179	4.1667	4.0423
Discomfort	2.8898	5.5556	4.2227
Health perceptions	3.3792	4.1667	3.7730
Coping	4.2505	8.3333	6.2919
Stress impact	4.2640	2.7778	3.5209
Total score	2.3709	6.7262	4.5485

**Table 4:** Logistic regression analysis of the association between the daily activity domain and changes to each syndrome element scores.

Independent variables	*B*	*S*. *E*.	Wald	df	*P*	Exp(*B*)
Liver	0.011	0.117	0.009	1	0.926	1.011
Stomach	0.155	0.152	1.040	1	0.308	1.167
Heat	0.037	0.128	0.085	1	0.771	1.038
Spleen-dampness	0.538	0.242	4.926	1	0.026	1.712
Qi deficiency	3.116	0.438	50.563	1	<0.001	22.556
Qi stagnation	0.012	0.084	0.020	1	0.888	1.012
Constant	−2.451	0.558	19.313	1	<0.001	0.086

**Table 5:** Logistic regression analysis of the association between the anxiety domain and changes to each syndrome element scores.

Independent variables	*B*	*S*. *E*.	Wald	df	*P*	Exp(*B*)
Liver	2.208	0.364	36.804	1	<0.001	9.100
Stomach	0.218	0.196	1.235	1	0.266	1.243
Heat	0.342	0.151	5.114	1	0.024	1.408
Spleen-dampness	0.188	0.196	0.923	1	0.337	1.207
Qi deficiency	0.066	0.203	0.107	1	0.744	1.069
Qi stagnation	2.214	0.379	34.092	1	<0.001	9.154
Constant	−13.897	2.033	46.715	1	<0.001	0.000

**Table 6:** Logistic regression analysis of the association between the diet domain and changes to each syndrome element scores.

Independent variables	*B*	*S*. *E*.	Wald	df	*P*	Exp(*B*)
Liver	0.277	0.101	7.458	1	0.006	0.277
Stomach	0.664	0.212	9.778	1	0.002	0.664
Heat	0.138	0.093	2.218	1	0.136	0.138
Spleen-dampness	0.191	0.133	2.075	1	0.150	0.191
Qi deficiency	0.353	0.136	6.767	1	0.009	0.353
Qi stagnation	0.035	0.065	0.284	1	0.594	0.035
Constant	−2.56	0.471	29.515	1	<0.001	−2.560

**Table 7:** Logistic regression analysis of the association between the sleep domain and changes to each syndrome element scores.

Independent variables	*B*	*S*. *E*.	Wald	df	*P*	Exp(*B*)
Liver	0.220	0.098	5.027	1	0.025	1.247
Stomach	0.426	0.187	5.198	1	0.023	1.532
Heat	0.238	0.094	6.386	1	0.012	1.269
Spleen-dampness	0.808	0.198	16.638	1	<0.001	2.242
Qi deficiency	0.027	0.12	0.051	1	0.822	1.027
Qi stagnation	−0.005	0.067	0.006	1	0.936	0.995
Constant	−2.266	0.452	25.081	1	<0.001	0.104

**Table 8:** Logistic regression analysis of the association between the discomfort domain and changes to each syndrome element scores.

Independent variables	*B*	*S*. *E*.	Wald	df	*P*	Exp(*B*)
Liver	0.001	0.111	0.000	1	0.992	1.001
Stomach	6.617	0.986	45.063	1	<0.001	747.861
Heat	0.434	0.129	11.303	1	0.001	1.544
Spleen-dampness	0.088	0.134	0.429	1	0.512	1.092
Qi deficiency	0.188	0.167	1.271	1	0.260	1.206
Qi stagnation	−0.161	0.094	2.948	1	0.086	0.851
Constant	−3.129	0.584	28.657	1	<0.001	0.044

**Table 9:** Logistic regression analysis of the association between the health perception's domain and changes to each syndrome element scores.

Independent variables	*B*	*S*. *E*.	Wald	df	*P*	Exp(*B*)
Liver	0.013	0.08	0.025	1	0.874	1.013
Stomach	0.284	0.148	3.698	1	0.054	1.329
Heat	0.295	0.097	9.325	1	0.002	1.343
Spleen-dampness	0.123	0.125	0.965	1	0.326	1.131
Qi deficiency	0.235	0.129	3.294	1	0.070	1.264
Qi stagnation	0.089	0.068	1.702	1	0.192	1.093
Constant	−0.691	0.354	3.810	1	0.051	0.501

**Table 10:** Logistic regression analysis of the association between the coping domain and changes to each syndrome element scores.

Independent variables	*B*	*S*. *E*.	Wald	df	*P*	Exp(*B*)
Liver	0.083	0.113	0.542	1	0.462	1.087
Stomach	0.343	0.175	3.842	1	0.050	1.409
Heat	2.157	0.306	49.814	1	<0.001	8.643
Spleen-dampness	0.325	0.205	2.525	1	0.112	1.384
Qi deficiency	−0.062	0.153	0.166	1	0.684	0.940
Qi stagnation	0.104	0.094	1.237	1	0.266	1.110
Constant	−2.110	0.51	17.097	1	<0.001	0.121

**Table 11:** Logistic regression analysis of the association between the stress domain and changes to each syndrome element scores.

Independent variables	*B*	*S*. *E*.	Wald	df	*P*	Exp(*B*)
Liver	0.165	0.110	2.235	1	0.135	1.180
Stomach	0.254	0.182	1.942	1	0.163	1.289
Heat	0.221	0.097	5.168	1	0.023	1.248
Spleen-dampness	0.219	0.13	2.821	1	0.093	1.244
Qi deficiency	0.066	0.133	0.249	1	0.618	1.068
Qi stagnation	−0.045	0.064	0.48	1	0.488	0.956
Constant	−2.569	0.511	25.228	1	<0.001	0.077

**Table 12:** Logistic regression analysis of the association between the total scores domain and changes to each syndrome element scores.

Independent variables	*B*	*S*. *E*.	Wald	df	*P*	Exp(*B*)
Liver	0.397	0.119	11.046	1	0.001	1.487
Stomach	0.463	0.181	6.544	1	0.011	1.589
Heat	0.493	0.116	18.081	1	<0.001	1.637
Spleen-dampness	1.415	0.286	24.469	1	<0.001	4.118
Qi deficiency	0.370	0.159	5.429	1	0.020	1.448
Qi stagnation	0.191	0.092	4.293	1	0.038	1.210
Constant	−3.696	0.59	39.189	1	<0.001	0.025

## Data Availability

The data used to support the findings of this study are available from the corresponding author upon request. Requests for data, (6/12 months) after publication of this article, will be considered by the corresponding author.

## Supplementary Materials

Supplementary Materials Figure 1. The receiver operating characteristic (ROC) curve of every syndrome. Clinicians' syndrome differentiation results were divided into syndrome element forms, according to the theory of syndromes, and used as state variables. The CAT model diagnosis results were used as test variables. Table 1. ROC curve analysis results of CAT model at baseline. Table 2. ROC curve analysis results of CAT model at follow-up. According to the results, the area under the curve (AUC) for the CAT model was of >0.8 both at baseline and follow-up, which are indicative of high model accuracy.
