# Supplementary material for: Interpretation of a Quantitative Diagnosis Model of Traditional Chinese Medicine Syndromes Based on Computer Adaptive Testing
Source: Evid Based Complement Alternat Med. 2022 Jun 30;2022:3203158. doi: 10.1155/2022/3203158 (PMC9262526; doi:10.1155/2022/3203158)
Supplement: Supplementary Materials — Figure 1. The receiver operating characteristic (ROC) curve of every syndrome. Clinicians' syndrome differentiation results were divided into syndrome element forms, according to the theory of syndromes, and used as state variables. The CAT model diagnosis results were used as test variables. Table 1. ROC curve analysis results of CAT model at baseline. Table 2. ROC curve analysis results of CAT model at follow-up. According to the results, the area under the curve (AUC) for the CAT model was of >0.8 both at baseline and follow-up, which are indicative of high model accuracy. [file 3203158.f1.doc]

Appendix I


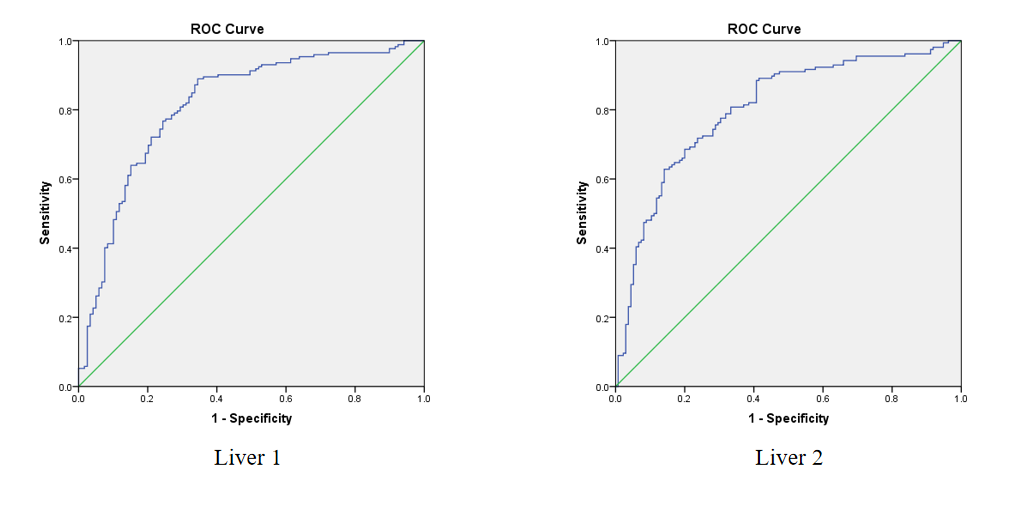


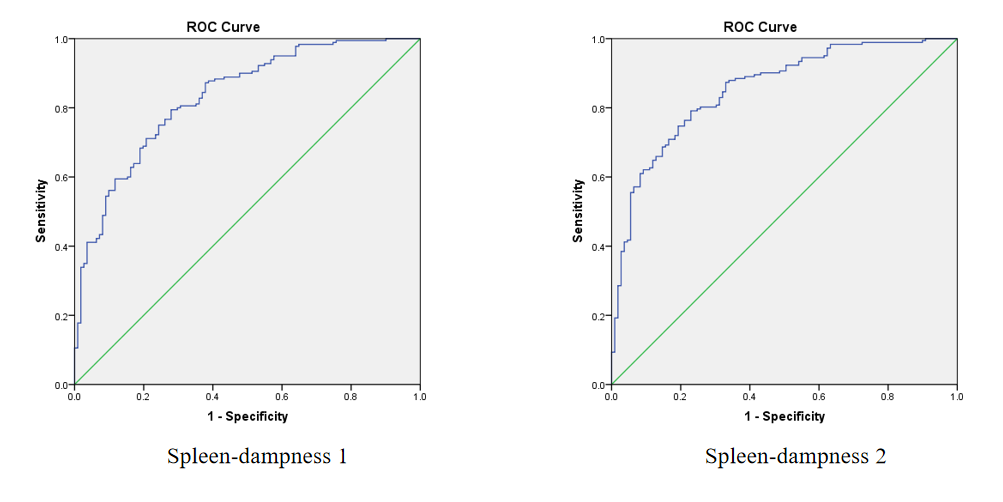


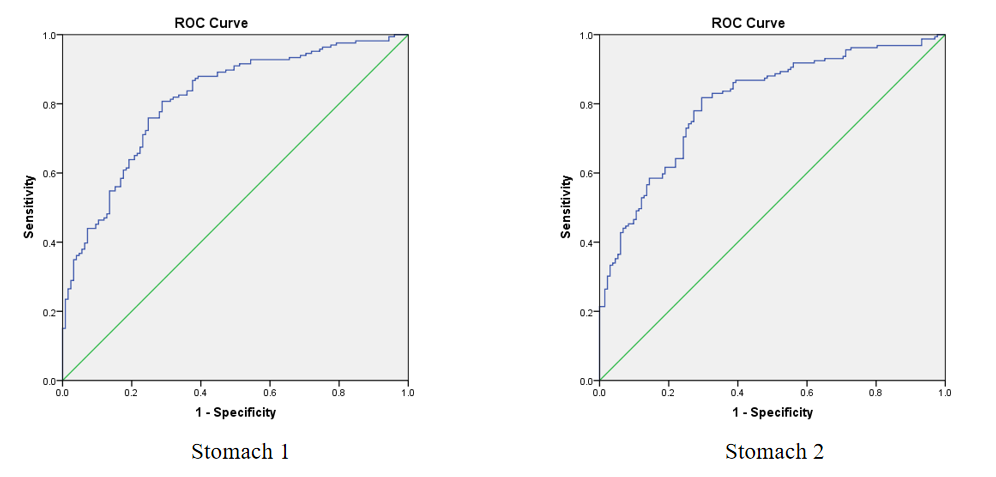


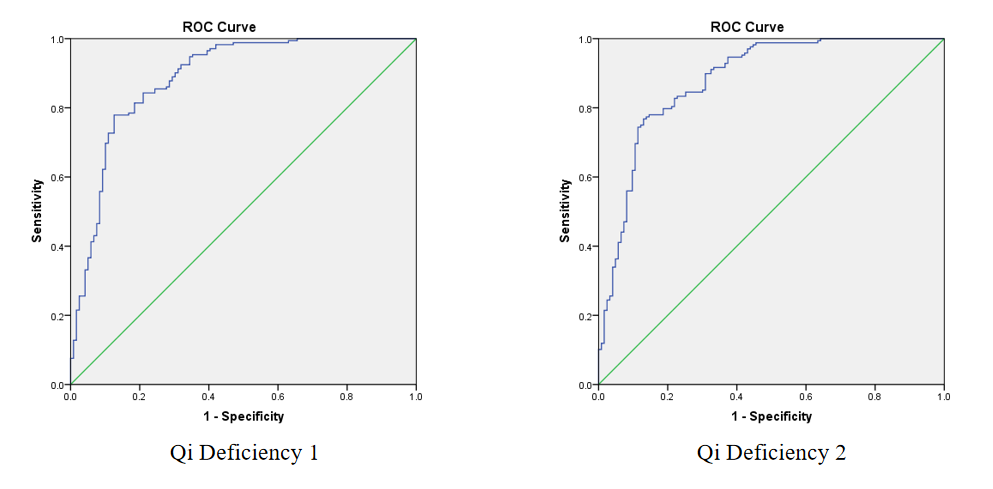


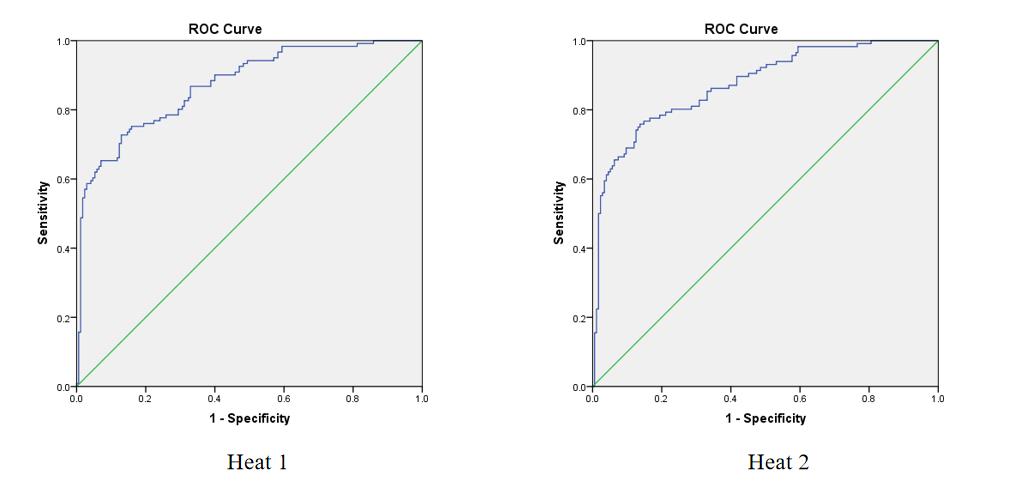


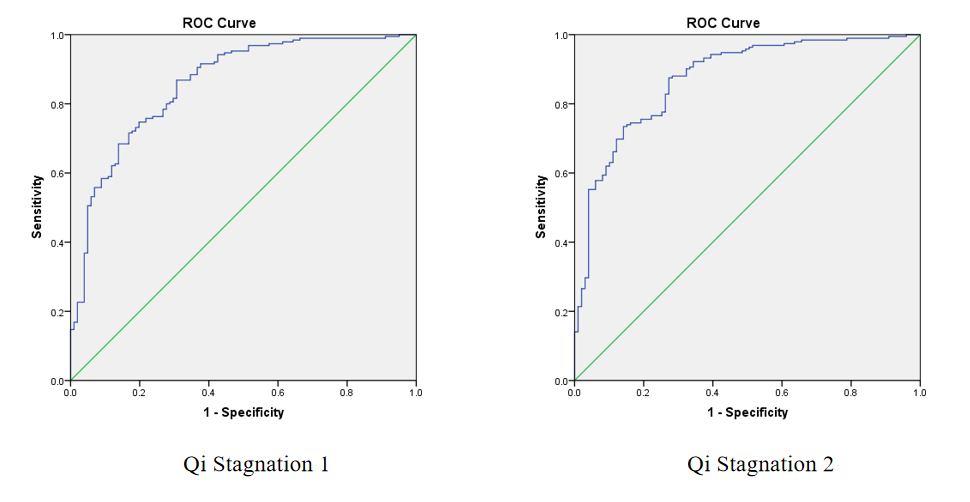


**Figure1:** The receiver operating characteristic (ROC) curve of every syndrome.

| **Table 1**  ROC curve analysis results of CAT model at baseline | | | | |
| --- | --- | --- | --- | --- |
| Syndrome element | AUC | SE | P | 95%CI |
| Liver 1 | 0.815 | 0.026 | ＜0.001 | （0.764，0.867） |
| Stomach 1 | 0.812 | 0.025 | ＜0.001 | （0.763，0.861） |
| Spleen-dampness 1 | 0.832 | 0.024 | ＜0.001 | （0.785，0.879） |
| Qi Deficiency 1 | 0.886 | 0.021 | ＜0.001 | （0.845，0.927） |
| Heat 1 | 0.872 | 0.021 | ＜0.001 | （0.831，0.913） |
| Qi Stagnation 1 | 0.858 | 0.023 | ＜0.001 | （0.812，0.903） |

| **Table 2** ROC curve analysis results of CAT model at follow-up | | | | |
| --- | --- | --- | --- | --- |
| Syndrome element | AUC | SE | P | 95%CI |
| Liver 2 | 0.804 | 0.026 | ＜0.001 | （0.753，0.855） |
| Stomach 2 | 0.806 | 0.025 | ＜0.001 | （0.756，0.856） |
| Spleen-dampness 2 | 0.854 | 0.022 | ＜0.001 | （0.810，0.898） |
| Qi Deficiency 2 | 0.881 | 0.021 | ＜0.001 | （0.840，0.922） |
| Heat 2 | 0.874 | 0.021 | ＜0.001 | （0.833，0.916） |
| Qi Stagnation 2 | 0.873 | 0.022 | ＜0.001 | （0.830，0.916） |
